# Salvage Surgery in Recurrent Oral Squamous Cell Carcinoma

**DOI:** 10.3389/froh.2021.815606

**Published:** 2022-01-28

**Authors:** K. S. Rathan Shetty, Vinayak Kurle, P. Greeshma, Veena B. Ganga, Samskruthi P. Murthy, Siddappa K. Thammaiah, P. Krishna Prasad, Purushottham Chavan, Rajshekar Halkud, R. Krishnappa

**Affiliations:** Department of Head and Neck Oncology, Kidwai Memorial Institute of Oncology, Bengaluru, India

**Keywords:** recurrent, salvage surgery, oral cancer, outcome, decision making

## Abstract

More than half of patients with oral cancer recur even after multimodality treatment and recurrent oral cancers carry a poorer prognosis when compared to other sites of head and neck. The best survival outcome in a recurrent setting is achieved by salvage surgery; however, objective criteria to select an ideal candidate for salvage surgery is difficult to frame, as the outcome depends on various treatment-, tumor-, and patient-related factors. The following is summarizes various tumor- and treatment-related factors that guide our decision-making to optimize oncologic and functional outcomes in surgical salvage for recurrent oral cancers. Short disease-free interval, advanced tumor stage (recurrent and primary), extracapsular spread and positive tumor margins in a recurrent tumor, regional recurrence, and multimodality treatment of primary tumor all portend worse outcomes after surgical salvage. Quality of life after surgical intervention has shown improvement over 1 year with a drastic drop in pain scores. Various trials are underway evaluating the combination of immunotherapy and surgical salvage in recurrent head and neck tumors, including oral cavity, which may widen our indications for salvage surgery with improved survival and preserved organ function.

## Introduction

Oral cavity squamous cell carcinoma (OCSCC) is one of the common cancers globally and their oncologic outcome has remained stable over decades [1]. Tumor relapses occur in 25–30% of cases in early-stage OCSCC and 50–60% of cases in advanced OCSCC [2–5]. Managing relapsed tumors is difficult due to exhausted treatment options, as most of the patients are previously heavily treated with surgery with/without adjuvant therapy. These tumors represent resistant clones of cells that have escaped treatment and, hence, have guarded prognosis [6]. Immunotherapy has shown promising results in recurrent/metastatic tumors of head and neck (R/M HN) and has emerged as the first-line treatment modality for the recurrent disease that is not amenable for curative-intent management [7]. Specific guidelines are framed regarding the choice of systemic therapy in R/M HN setting, which considers previous treatment, performance status of the patient, and combined positive score (CPS) [8].

Tumor resection with adequate margins and adjuvant therapy form the main treatment modality in both the primary and recurrent setting in OCSCC and offer the best survival benefit [6, 9–11]. However, no specific criteria exist to precisely select the patients for surgical salvage (SS). The available literature on recurrent oral cancers (ReOC) is retrospective and is commonly represented along with other head and neck sites. Most patients with OCSCC have field cancerization, making differentiation between second primary and recurrent tumor hard and are often reported together. Objective criteria to accurately select patients for SS are difficult to frame, as the success of SS is based on numerous factors and vary from patient to patient. Apart from the anatomical and functional constraints, poorly vascularized bed, heavily pretreated areas of healing, performance status, comorbidities, previous treatment morbidity, and various recurrent tumor factors also play a crucial role in decision-making.

We comprehensively reviewed literature from Medline and Embase databases to include studies evaluating outcomes on salvage surgery in ReOC. The following review summarizes tumor- and treatment-related factors reported in these studies that guide our decision-making to optimize oncologic and functional outcomes in SS for ReOC.

## Pattern of Recurrence

Two-thirds of patients with head and neck cancer recur with most presenting in <24 months from completion of treatment for primary tumor [6, 11]. The rates of local, regional, and locoregional recurrences in OCSCC are 31.2–62.6%, 24–51.1%, and 4.1–16.3%, respectively [12–18]. Knowledge and understanding of recurrence pattern are of substantial importance, as it aids in early detection, assessment of resectability, and preoperative planning.

### Local Relapse

Previous surgery and/or radiation therapy (RT) open planes of tumor barrier and bring changes in anatomical orientation, which may render locally recurrent tumor unresectable even when detected early. Tongue cancers migrate longitudinally along muscles fibers and lymphovascular planes and previous resection may result in early involvement of mylohyoid muscle, which portends a worse prognosis [19, 20]. Primary buccal cancers involving masticator space, most often present with local recurrence above supramandibular notch, bringing recurrent tumor close to vital areas such as foramen ovale, pterygoid plates, and cavernous sinus making resection more complicated [21]. Unexpected local recurrences in buccal cancer are seen secondary to retrograde infiltration of the nerve [21, 22]. Local relapse commonly occurs at the anastomotic site of previous reconstructive flap within flap tissue or a remote oral cavity site [23].

Local recurrences carry better prognosis after SS than regional/locoregional recurrences [24]. Sessions et al. demonstrated that SS for recurrent tongue (local) cancer had a better 5-year cumulative survival rate than those with regional recurrence (48.6 vs. 30.5%, respectively) [25]. Although, Mucke et al. aimed to investigate the impact of recurrence interval on survival, authors also found that patients with local recurrence fared better than those with regional recurrence [5-year overall survival (OS) 37.5 vs. 21.5%, respectively] [26]. Positive surgical margin in SS is a predictor of poor prognosis [27, 28]. Use of frozen section to evaluate margins intraoperatively is advisable, especially in a salvage setting, as most of the surrounding tissue is fibrotic and makes margin assessment difficult. The frozen section is assessed from resected specimen rather than tumor bed [29].

### Regional Relapse

Regional recurrences are commonly seen in level II nodal region [30]. Unusual regional recurrences occur in ReOC at intraparotid, prelaryngeal, and retropharyngeal sites [30, 31]. However, patients with unusual regional recurrences have a poorer SS success rate (21.7 vs. 68.8%, *p* < 0.001) and 5-year disease-specific survival (DSS) (23.8 vs. 60.8%, *p* < 0.001) than those without unusual regional recurrences [31]. Mizrachi et al. evaluated 1,302 cases of oral cancer for regional recurrence in RT-naïve patients. 15% of patients developed regional recurrence and most (87%) patients underwent salvage treatment [32]. Patients who underwent SS with adjuvant chemoradiation (CTRT) therapy had better DSS than surgery alone or non-SS. Regional recurrence is best managed with surgery with or without adjuvant therapy. Another study demonstrated that regional relapse in a previously treated neck and with short disease-free interval (DFI) was associated with a significant reduction in survival [33].

## Disease-Free Interval

Oral cavity examination has an easier access when compared to other head and neck sites. In a recurrent setting, tissue changes from previous treatment preclude recurrent tumor identification. The onset of new or persistent symptoms should alert the treating surgeon for active intervention. Surveillance with CT) or MRI scan is considered as standard protocol in most centers. Diffusion-weighted MRI is invaluable in identifying recurrent tumors even in asymptomatic patients. PET/CT surveillance can also be used and a short time to positive PET/CT carries poorer outcomes in ReOC [34].

Disease-free interval is defined as the time interval between completion of primary treatment and occurrence of recurrent tumor. Various studies have predicted that short DFI is associated with poorer survival and is an independent predictor of outcome in ReOC [15, 26, 34]. There is heterogeneity in defining optimal cutoff for early vs. late recurrence. Two studies that considered 18 months as optimal cutoff to define early vs. late recurrence have reported lower OS with recurrences <18 months when compared to recurrences occurring >18 months (20.5 vs. 42.3% and 27.6 vs. 38.2%, respectively) [26, 34]. Liao et al. reported an optimal cutoff for interval to relapse as 10 months and late recurrence was associated with significantly better 5-year DSS and OS (*p* < 0.0001) [15]. A recent study by Hosni et al. has shown that early recurrence in patients with OCSCC treated with surgical resection and before initiating planned postoperative RT is seen in 15% of cases [35]. They noted that patients with oral tongue primary, microscopic positive margin, pathological tumor (pT) stage III/IV, and pathological nodal (pN) stage II/III were associated with early recurrence on multivariate analysis. The 3-year OS significantly (*p* = 0.001) dropped from 71% (95% CI, 67–75%) to 41% (95% CI, 30–56%) in patients with no early recurrence to those who recurred before planning adjuvant therapy, respectively. Hence, an early recurrence carries a worse prognosis and must be factored during decision-making and patient counseling.

## Survival Predictor Scores

Various staging and risk stratifications of the patient have been proposed to aid in choosing the right patient for salvage treatment [5, 20, 36–38]. Yueh et al. proposed the first prognostic staging system for 308 persistent, recurrent, and second primary tumors of the oral cavity and oropharynx [36]. They proposed a composite 4-stage system with three variables, namely, tumor nodal metastasis (TNM) staging of recurrent tumor, weight loss (no weight loss, <20%, and ≥20%), and deep muscle invasion (i.e., mylohyoid/constrictor muscle involvement for oral cavity and pharyngeal tumors, respectively). For patients with T2N0M0, no weight loss and for patients with T2N0M0, <20% weight loss, 1-year survival rate was 88.2 and 71.9%, respectively, and 1-year survival rate dropped to 32.6% when weight loss was ≥ 20% and/or with deep muscle invasion. However, for patients with metastasis, the 1-year survival rate was 4.2%, regardless of weight loss. An important finding was that persistent tumors fare worse than recurrent tumors, which, in turn, fare worse than second primary tumors.

Lacy et al. also proposed a new staging system for recurrent oral cavity and oropharyngeal cancers, which included primary tumor (initial) TNM staging and extent of recurrence (local, regional, and distant recurrences) [20]. The 2-year survival rate was 54 and 41% for stage I (initial TNM stage I with local recurrence) and stage II (initial TNM stage I with regional recurrence or stage II with local recurrence), respectively, and 18 and 3% of 2-year survival rate for stage III (initial TNM stages III or IV with local or regional recurrence) and stage IV (any patient with distant metastasis), respectively. The following study highlights the fact that local recurrence has better outcomes than regional recurrent tumors. Sun et al. also proposed a staging system that incorporated both the primary and recurrent tumor staging with OCSCC [10]. Authors reported a significant survival difference between the proposed stages (*p* = 0.000) and those with locoregional recurrences, multiple nodes at recurrence and incompletely excised recurrent tumor fared worse. Tam et al. investigated survival outcome in ReOC. On recursive partition analysis, they identified the three risk groups: (1) the high-risk group (patients who received adjuvant CTRT/RT after initial therapy), (2) the intermediate-risk group (previous surgery alone and age ≥ 62 years), and (3) the low-risk group (previous surgery alone and age <62 years) with 5-year OS rate of 10, 39, and 74%, respectively [37]. Authors concluded that patients belonging to the high-risk group must be considered for noncurative intent treatment. Although risk stratification aids surgeon in choosing the right patient and counseling them regarding oncologic outcomes, they are not validated on external data and may not be applicable universally.

## Adjuvant RT After SS

The indications for adjuvant therapy after SS in ReOC remain the same as the primary setting [39, 40]. In radiation-naïve patients, adjuvant radiation does not pose any specific morbidity. However, reirradiation (ReRT) must be suggested with caution in patients who received RT for primary tumors. Tumors that recur at the site of the previous radiation field after >50 Gy of total radiation dose within 6 months are considered to have radioresistant tumor [41] and the survival benefit with ReRT must be critically balanced against ReRT induced adverse effects. With conformal radiotherapy techniques such as intensity-modulated radiotherapy (IMRT) and volumetric modulated arch therapy, ReRT is now possible. Janot et al. conducted a randomized controlled trial to compare ReRT concurrent with chemotherapy vs. observation after SS in patients with recurrent head and neck carcinoma [42]. The trial reported a significantly improved DSS with adjuvant ReRT [hazards ratio (HR) 1.68, 95% CI, 1.13–2.50; *p* = 0.01] when compared to no ReRT. However, acute toxicity (≥grade 3) and late toxicity (≥grade 3) were seen in 28 and 39% of patients in ReRT arm, respectively. The RT technique used was three-dimensional (3D) conformal radiotherapy and IMRT was not used in the trial. Pathological risk features in the RT arm of the trial were suspicious/involved margin (22%), vascular emboli/perineural infiltration/diffuse infiltration (54%), and capsular rupture (70%). The trial included 18% of ReOC cases. May et al. suggested postoperative ReRT in recurrent head and neck cancers with positive surgical margin and perineural invasion, based on negative impact on progression-free survival when postoperative ReRT was omitted in cases with positive margin (HR: 8.894, 95% CI: 1.742–45.403) and perineural invasion (HR: 3.391, 95% CI: 1.140–10.089) [43].

Decisions regarding radiation dose and fractionation for adjuvant ReRT may vary from center to center. A recent retrospective multi-institutional study evaluated the efficacy of IMRT ReRT in 505 patients with recurrent/second primary tumor of head and neck (16.6% oral cavity subsite) [44]. This study concluded that doses 50 to 66 Gy were adequate after removing gross disease. The acute and late toxicity rates were 22.1 and 16.7%, respectively. Hyperfractionation and elective neck irradiation were not associated with oncologic benefit. Adjuvant RT must be considered after SS when feasible to achieve best survival.

## Survival After SS

Radiation-naïve patients with ReOC after SS have a 5-year OS, recurrence-free survival, and locoregional control rates of 59, 60, and 74%, respectively, and seem similar to locally advanced patients with nonrecurrent oral cancer treated with multimodality therapy [3, 45]. A study compared outcomes of ReOC after early OSCC (RT naïve) vs. advanced OSCC who received multimodality treatment with no recurrence. They showed that early OSCC with recurrence fared worse than advanced OSCC that did not recur [46]. However, the study is inherently biased as advanced OSCC with no recurrence represent favorable tumor biology than early tumors with recurrence. Various studies (as per comprehensive literature search) published after 2010, which have evaluated survival outcomes after SS in ReOC, are represented in Table 1.

**Table 1:** Studies evaluating survival outcomes after salvage surgery for recurrent oral cancer in the recent decades.

**Year of publication and authors**	**Number of patients**	**Treatment of primary tumor**	**Treatment of recurrent tumor**	**Survival outcome after salvage surgery**	**Factors adversely affecting survival after salvage surgery**
2010					
Agra et al. [47]	41^*^	NA	Salvage surgery ± adjuvant therapy	3-year cancer specific survival 20%	• <6 months of disease free interval
Kernohan et al. [17]	77	Surgery, Radiotherapy, Surgery ± Adjuvant therapy	Salvage surgery ± adjuvant therapy	2-Year disease specific survival 78% for local recurrence and 20% for regional recurrence treated with multimodality treatment	• Initial combined modality treatment • Shorter time to recurrence
2016					
Goto et al. [48]	69	Surgery, Radiotherapy, Surgery ± Adjuvant therapy	Salvage surgery ± adjuvant therapy	5 year Overall survival 79 vs 44% 78 vs 37% 86 vs 48%	• No recurrent nodal disease Vs > 2 recurrent nodal metastasis • No Extracapsular spread Vs + extra capsular spread at recurrence • Recurrent tumor stage I/II Vs III/IV • Short disease free interval <12 months
Horn et al. [49]	32	Surgery, Radiotherapy, Surgery ± Adjuvant therapy	Salvage surgery ± adjuvant therapy	2-year Overall survival and disease free survival 37.8% and 30.9% respectively	• Microscopic positive margins at salvage surgery
Liu et al. [50]	27	Surgery alone	Salvage surgery ± adjuvant therapy, radiation therapy	5- year overall survival and Disease specific survival was 50% and 61% respectively	• Regional recurrences
2017					
Tam et al. [37]	59	Surgery ± adjuvant therapy	Salvage surgery ± adjuvant therapy, radiation therapy, no salvage therapy	5-year overall survival after salvage surgery 43%	• Multimodality treatment for primary tumor
2018					
Matsuura et al. [28]	46	Surgery ± adjuvant therapy	Salvage surgery ± adjuvant therapy	Overall survival and Disease free survival was 31.7 and 35% respectively (Follow up maximum 61 months)	• Presence of regional recurrences and positive surgical margins during salvage surgery
Mizrachi et al. [32]	1,302^#^	Surgery for primary tumor. Neck dissection vs observation	Salvage surgery± adjuvant therapy, Chemoradiation therapy	1-year disease specific survival 72% for Salvage surgery with adjuvant therapy, 40% for surgery alone and 27% for non-surgical salvage	• Non-surgical salvage modality of treatment
Subramaniam et al. [51]	25	Surgery ± adjuvant therapy	Salvage surgery ± adjuvant therapy	5-year overall survival 12%	• Previous adjuvant therapy • adverse pathological features in recurrent tumor • <6 months of disease free interval
2019					
Weckx et al. [52]	159	Surgery ± adjuvant therapy	Salvage surgery, radiotherapy, chemoradiotherapy, palliative chemotherapy, palliative radiotherapy, supportive therapy	5-year and 10-year overall survival 66 and 56% respectively	• Early time to recurrence • Positive margin and extracapsular spread in recurrent tumor
2020					
Chung et al. [53]	73	Surgery ± adjuvant therapy, Chemoradiation therapy	Surgery + chemoradiation therapy, others	5-year overall, locoregional failure free and disease free survival were 54.8, 58.9 and 49.3%	• Advanced nodal stage of primary tumor • Multimodality treatment for primary tumor • Locoregional recurrence • Advanced recurrent tumor stage • Short disease free interval <8 months
2021					
Szewczyk et al. [54]	108	Surgery ± adjuvant therapy	Surgery, palliative care	5-year overall survival 58%	• Positive surgical margins after salvage surgery
Nandy et al. [55]	168	Surgery ± adjuvant therapy	Surgery ± adjuvant therapy, Neoadjuvant chemotherapy followed by Surgery ± adjuvant therapy	2-year and 3-year overall survival were 37.6 and 21.8% respectively and 2-year and 3-year disease free survival were 26.2 and 14.7% respectively.	• Advanced stage and multimodality treatment of initial tumor • Regional recurrence and perineural invasion in recurrent tumor.
Yosefof et al. [56]	55^#^	Surgery ± adjuvant therapy	Surgery ± adjuvant therapy, chemoradiotherapy	5-year disease specific survival 46.7% and overall survival 35.3%	• <10 months of disease free interval
Chen et al. [57]	556	NA	Salvage surgery, radiotherapy, palliative chemotherapy, palliative radiotherapy.	2-year disease free survival with/without pathological risk factors 32.4% and 77.2% respectively and 2-year overall survival with/without pathological risk factors 58.4 and 89.2% respectively	• Pathological risk factors i.e. positive margins and extra capsular spread of recurrent tumor

* *Rerecurrent oral cancer*.

# *Nodal recurrent tumor in oral cancer. NA, not reported. ±, with or without*.

Extracapsular spread (ECS) is a strong predictor of systemic spread [48]. Presence of ECS in the primary tumor is a known predictor of poor outcomes; however, its presence in primary tumor has also been associated with poor survival even after salvage of ReOC, regardless of TNM staging or DFI [52]. Tumor thickness of >10 mm in a recurrent tumor is associated with shorter OS [15]. Adverse pathological features such as lymphovascular and perineural invasion are associated with a worse prognosis. Primary and recurrent tumor stages have been proven to be poor prognosticators for survival after SS [47, 51, 53, 58].

50–60% of patients with recurrent oral cavity and oropharyngeal tumors after their SS are at a greater risk of rerecurrence and <20% develop distant metastasis on follow-up [47]. The clinical staging had no bearing on the rate of rerecurrence in these patients. DFI between first and second recurrence is vital, as second recurrences occurring in <6 months had a 3-year cancer-specific survival of 0% [50]. Hence, patients presenting with rerecurrence and a short DFI are considered for palliative intent management. Second salvage surgery must be reserved for motivated patients with good performance status and with >6 months of DFI only.

## Reconstruction

In 1970's, Gilbert and Kagan reported that only 18.3% of recurrent oral and oropharyngeal tumors are surgical salvageable due to limitations related to reconstruction [59]. However, with advances in free flap reconstructions and microvascular techniques, most defects can be reconstructed with success rates of >90% in most series [60].

The overall complication rates are 18–24.1% in the recurrent setting [49, 61, 62]. Previous treatment details such as the extent of resection(especially mandible/maxilla), available vessels for microvascular anastomosis, radiation dose, time since RT completion, comorbidities, smoking, and preexisting wound infection all play a critical role in preoperative planning [63, 64]. Prior radiotherapy has shown to reduce graft bed vascularization continuously with increasing total RT dose and time after RT [65].

Microvascular reconstruction has shown a success rate of 92–96.8% in the ReOC setting [61, 66]. Selecting an appropriate recipient vessel in the neck is crucial for a successful outcome. Recipient vessels are prone to spasm and anastomotic complications due to inadvertent injury resulting from difficult dissection through fibrotic bed. Offodile et al. studied outcomes of sequential microvascular reconstruction for recurrent and second primary oral cancers [67]. The incidence of free flap failure was 1.6 and 8.1% in second and third sequential reconstructions. The duration of hospital stay and re-exploration for venous occlusion were higher with subsequent free flaps. This study showed that anterolateral thigh (ALT) flap was the most common flap used and free fibula flap was commonly used at first recurrence, highlighting that most salvage resection involved bone. In salvage settings, the microvascular anastomosis to contralateral superior thyroid/facial vessels and ipsilateral superficial temporal/transverse cervical vessels were frequent [67].

The lure to perform locoregional flaps in ReOC when survival outcome is limited must be weighed against the higher incidence of complications and wound dehiscence. Pectoralis major myocutaneous flap and extended vertical lower trapezius flaps have been described to reconstruct heavily pretreated defects in ReOC with acceptable outcomes [68, 69]. However, the requisite volume or tissue component required to restore integrity and function can be easily achieved by free flaps.

Many factors create a hostile environment for normal wound healing in a salvage setting; hence, a comprehensive approach, which includes intricate preoperative planning and preparation of the patient, is vital to achieve favorable outcome.

## Quality of Life (QOL) After SS

Extensive resection and radiation in ReOC are known to cause significant morbidity. A recent study by Horn et al. is one of the first to study quality of life (QOL) measures after SS in ReOC [70]. The QOL was assessed according to the European Organization for Research and Treatment of Cancer (EORTC) and utilized general questionnaire QLQ-C30 and specific tool QLQ-H&N35 questionnaires. They reported significant drop in global QOL during the first 3 months after SS; however, QOL recovered to baseline over a year. Other domains such as role functioning, emotional functioning, and physical functioning showed decrease in scores for first 3 months, but recovered over a year. Swallowing and speech items were rated the worst, nevertheless mean scores increased after a year. An important finding in this study was significant reduction in pain scores at 3 months after SS and remained way below baseline values even after a year. SS in ReOC is justified, as most domains in QOL improved over a year with significant reduction in pain scores.

## Future Perspective

Although SS offers the best chance of survival in ReOC, the oncologic outcome is still poorer, especially when compared to other subsites. Various trials are underway to assess the feasibility of immunotherapy in recurrent head and neck tumors (including oral cavity) planned for SS in neoadjuvant and adjuvant settings (Table 2). With the emergence of further trials testing the impact of combining immunotherapy and SS therapy in recurrent tumors of head and neck, it may be possible to widen the indications for SS and optimize oncologic outcome without compromising organ function and preserve QOL.

**Table 2:** Illustrative listing of clinical trials examining impact of combining immunotherapy in salvage surgery setting in recurrent head and neck cancers.

**Sl No**	**NCT number**	**Title**	**Phase**	**Outcome measure**	**Status**
1	NCT04754321	Pembrolizumab and Radiation therapy before and during surgery for treatment of persistent or recurrent head and neck cancer	I	Incidence of adverse events Progression free survival Overall survival Local control rate Health related quality of life	Not yet recruiting
2	NCT04671667	Testing what happens when an immunotherapy drug is added to radiation or given by itself compared to usual treatment of chemotherapy with radiation after surgery for recurrent head and neck squamous cell carcinoma	II	Incidence of adverse events Overall survival Disease free survival	Recruiting
3	NCT04188951	A pilot study of immunotherapy as consolidation therapy for patients with recurrent head and neck cancer	I	Toxicity rates Determine feasibility of immunotherapy after salvage surgery in recurrent head and neck tumors	Recruiting
4	NCT03565783	Cemiplimab in treating participants with recurrent stage III-IV Head and neck squamous cell cancer before surgery	II	Overall response rate Time to recurrence Patterns of failure Disease-specific survival ∙Disease-free survival Overall survival	Recruiting
5	NCT03003637	ImmunoModulation by the Combination of Ipilimumab and Nivolumab Neoadjuvant to Surgery In Advanced or Recurrent Head and Neck Carcinoma	I / II	Phase I: number of patients that will endure a delay in surgery due to immunotherapy related toxicity Phase II: Tumor response to neoadjuvant immunotherapy in terms of tumor tissue pathological response at time of surgery compared to RECIST 1.1 (FDG-PET and perfusion and diffusion weighted MRI).	Completed
6	EudraCT 2017-0012711-17 [Gustave Roussy- France]	Adjuvant immunotherapy after salvage surgery in head and neck squamous cell carcinoma : phase 2 trial evaluating the efficacy and the toxicity of nivolumab alone, and of the combination nivolumab and ipilimumab	II	Toxicity profile Overall and Disease free survival Quality of life	On going

*RECISTs, response evaluation criteria in solid tumors; FDG-PET, fluorodeoxyglucose-PET*.

## Conclusion

Surgical salvage remains the standard of treatment in ReOC when resectable, as it offers the best chance of survival. Patients with advanced stage recurrent tumors and short DFI are not ideal candidates for SS. Various risk stratification systems have been proposed, which help in triaging the patient for curative intent modalities of salvage treatment. ECS and positive margins in recurrent settings portend poor outcomes and such patients must be advised adjuvant therapy when feasible. With the rising application of immunotherapy in managing recurrent head and neck tumors planned for SS, we can further make way to expand indications for SS with an improved oncologic outcome.

## Author Contributions

KS contributed to the conceptualization. SM and KS contributed to the writing original draft. SM, KS, VK, PG, VG, and PP contributed to the review and editing. ST, PC, RH, and RK contributed to the supervision of the manuscript. All authors contributed to the article and approved the submitted version.

## Conflict of Interest

The authors declare that the research was conducted in the absence of any commercial or financial relationships that could be construed as a potential conflict of interest.

## Publisher's Note

All claims expressed in this article are solely those of the authors and do not necessarily represent those of their affiliated organizations, or those of the publisher, the editors and the reviewers. Any product that may be evaluated in this article, or claim that may be made by its manufacturer, is not guaranteed or endorsed by the publisher.
